# A multimodal framework for extraction and fusion of satellite images and public health data

**DOI:** 10.1038/s41597-024-03366-1

**Published:** 2024-06-15

**Authors:** Dana Moukheiber, David Restrepo, Sebastián Andrés Cajas, María Patricia Arbeláez Montoya, Leo Anthony Celi, Kuan-Ting Kuo, Diego M. López, Lama Moukheiber, Mira Moukheiber, Sulaiman Moukheiber, Juan Sebastian Osorio-Valencia, Saptarshi Purkayastha, Atika Rahman Paddo, Chenwei Wu, Po-Chih Kuo

**Affiliations:** 1 Laboratory for Computational Physiology, Massachusetts Institute of Technology, Cambridge, Massachusetts USA; 2 Departamento de Telemática, Universidad del Cauca, Popayán, Cauca Colombia; 3 John A. Paulson School of Engineering and Applied Sciences, Harvard University, Boston, Massachusetts USA; 4 School of Computer Science, University College Dublin, Dublin, Ireland; 5 Grupo de Epidemiología, Facultad Nacional de Salud Pública, Universidad de Antioquia, Medellín, Colombia; 6 Department of Biostatistics, Harvard TH Chan School of Public Health, Boston, Massachusetts USA; 7 Department of Medicine, Beth Israel Deaconess Medical Center, Boston, Massachusetts USA; 8 Department of Computer Science, National Tsing Hua University, Hsinchu, Taiwan; 9 Department of Computer Science, Worcester Polytechnic Institute, Worcester, Massachusetts USA; 10 ScienteLab, Bogota, Cundinamarca Colombia; 11 Department of BioHealth Informatics, Indiana University Luddy School of Informatics, Computing, and Engineering, Indianapolis, Indiana USA; 12 Department of Electrical Engineering and Computer Science, University of Michigan, Ann Arbor, Michigan USA

**Keywords:** Environmental impact, Epidemiology

## Abstract

In low- and middle-income countries, the substantial costs associated with traditional data collection pose an obstacle to facilitating decision-making in the field of public health. Satellite imagery offers a potential solution, but the image extraction and analysis can be costly and requires specialized expertise. We introduce SatelliteBench, a scalable framework for satellite image extraction and vector embeddings generation. We also propose a novel multimodal fusion pipeline that utilizes a series of satellite imagery and metadata. The framework was evaluated generating a dataset with a collection of 12,636 images and embeddings accompanied by comprehensive metadata, from 81 municipalities in Colombia between 2016 and 2018. The dataset was then evaluated in 3 tasks: including dengue case prediction, poverty assessment, and access to education. The performance showcases the versatility and practicality of SatelliteBench, offering a reproducible, accessible and open tool to enhance decision-making in public health.

## Introduction

Traditional data collection and accessibility continue to pose challenges in low- and middle-income countries (LMIC) where the costs associated with data collection are high. Access to data for conducting analyses and predictions around problems such as poverty, health, and equity is crucial for achieving the sustainable development goals outlined by the United Nations^1^. On the other hand, unequal access to high-quality data with adequate temporality leads to disparities between countries with abundant resources and those with limited or moderate resources.

Given the lack of traditional data collection methods, it has been proposed to use alternative data sources, such as satellite images. The use of satellite images has proven to be a cost-effective means of obtaining real-time data access in countries where data is inaccessible due to social, environmental, or economic problems^2–4^. Satellite images have been used in applications across various domains, including environmental science and conservation for deforestation monitoring^5^, as well as in public health for measuring food security indices^6^, detecting poverty^7,8^ and predicting climate-sensitive diseases like dengue^9,10^, or malaria^11^ These applications are especially valuable in LMIC with limited resources, benefiting up to 128 economies and public health in the process^12–14^.

Although satellite images have proven to be a possible alternative, their analysis demands specialized expertise and incurs significant computational expenses for data processing and model utilization. Deep learning models such as those based on neural networks often require substantial computational resources, which are frequently costly and only available to a limited number of organizations worldwide. Therefore, there is an urgent need to develop highly optimized, lightweight, and affordable versions that can provide reliable performance on par with more costly alternatives. ShuffleNet^15^, ShuffleNet V2^16^, MobileNet^17^, squeezeNet^18^, and other optimization and pruning strategies have all produced comparable performance while using fewer resources.

It has been actively researched for decades to find vector embeddings with high correlations to the original data. Data has been effectively compressed using methods like Principal Components Analysis (PCA)^19^, UMAP^20^, T-SNE^21^, and more modern neural network models like VGG16^22^ EfficientNet-B0/B7^23^, ResNet^24^ and ViT^25^, maintaining critical information in less memory. Building such vector embeddings in satellite images holds the potential to provide valuable assistance to LMIC. These regions often face challenges in training complex models, yet the integration of diverse data sources can be pivotal in maximizing the utilization of available data. To understand patterns, provide solutions for health, and social determinants, the main challenges are specifically related to the collection of temporally aligned data.

In this work, we present SatelliteBench, a satellite imagery vector embedding framework tailored for bridging the data gap in regions where traditional data collection is economically unfeasible, with a focus on social determinants and climate-sensitive diseases. We also introduce a novel time series multimodal fusion pipeline based on the prevailing autoregressive prediction methodology, which heavily relies on historical epidemiological data that is often unavailable in real-life scenarios. Our scalable framework facilitates efficient and generalized satellite image extraction and vector embedding generation. The framework introduces a unique recursive de-noising algorithm to enhance the quality of the image by removing artifacts such as clouds or shadows. It also employs a cryptographic hash method to assess the quality and utilizes a ResNet-based Variational Autoencoder approach to extract satellite image embeddings compressing the information of the image.

The framework was tested by extracting satellite images of 81 municipalities in Colombia, South America, between 2016 and 2018, resulting in a total of 12,636 images^26^. Metadata for those municipalities was extracted using the local census and other satellites to gather temperature and precipitation values. The dataset was used in three use case scenarios: dengue prediction, poverty index assessment, and access to undergraduate and graduate education. In the case of dengue prediction, the proposed temporal data fusion pipeline was used to achieve better performance. To show the value of the image embeddings in a simpler task, a prediction of poverty and access to school was performed using a Support Vector Machine (SVM) model, which was selected due to the simplicity and time efficiency of the model. The models’ outstanding accuracy and compact footprint are what make this research significant. These models also have outstanding social advantages by providing a low-cost solution for countries with low and medium resources, are scalable, and are simple to modify for deployment throughout several cities and nations, where the need is greatest.

In summary, our contributions include:

Development of a framework for acquiring spatiotemporally aligned images along with relevant metadata. Creation of a versatile vector embedding extraction method for satellite images. Introduction of various time series data fusion models using a mutual information loss for multimodal data fusion. Evaluation across diverse use cases: Dengue, Poverty, and Education.

## Results

**Fig. 1:**
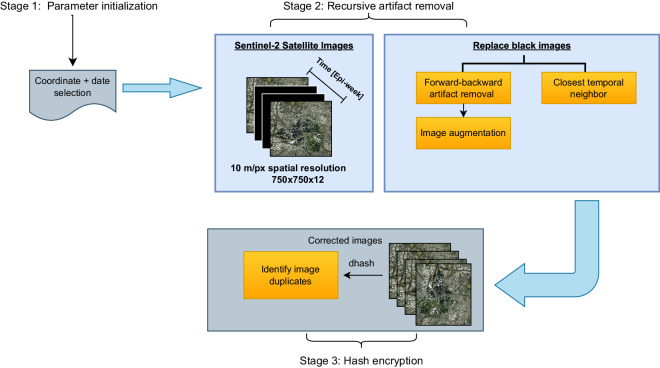
Satellite imagery extraction framework. A three-stage process to download satellite imagery with black image and noise correction.

As a result, a framework for the extraction of time-series from satellite images and its fusion with metadata (Fig. 1) was presented. Furthermore, a pipeline for the extraction of embeddings from the images wass proposed (Fig. 4c - Step 1). Finally, a data fusion model using time series was proposed and evaluated in the prediction of dengue cases (Fig. 4d - Step 2). The proposed framework was evaluated by generating a dataset of weekly satellite images in Colombia for 81 different cities between 2016 and 2018 with their corresponding metadata^26^.

The pipeline for the generation of embeddings and the pre-trained models are available online in the Hugging face’s profile of the organization (https://huggingface.co/MITCriticalData).

### Application scenarios

To show the potential of the satellite images, we illustrate how the research community could take advantage of our vector embeddings to produce cheap but yet accurate models. We illustrated this in 3 use case scenarios which are:

- **Poverty mapping:** SatelliteBench has the potential to substantially contribute to poverty alleviation efforts and inform targeted interventions, following the strategies proposed by Elvidge *et al*.^27^ for data-driven decision-making in development planning. Here we are able to achieve good performance using classic machine learning models like a Support Vector Machine (SVM) just concatenating in the input both, the embeddings and the metadata.
- **Geographical access to school:** SatelliteBench also holds immense promise in the realm of prediction access to school. Zhigang Han *et al*.^28^ underscores the significance of spatial data in the equitable distribution of geographical access to educational facilities. Leveraging our embeddings, which encapsulate geographical nuances and localized socio-economic dynamics, we offer an innovative approach to guide resource allocation decisions. Here we are able to achieve good performance using classic machine learning models like a Support Vector Machine (SVM) just concatenating in the input both, the embeddings and the metadata.
- **Dengue outbreak prediction:** Dengue is a re-emerging endemic disease that has infected over 3.9 billion people around 128 countries and throughout South Asia, South-East Asia, Africa, the Americas, the Western Pacific, and the Eastern Mediterranean regions^12,13,29^. Colombia is a country that has suffered from endemic Dengue outbreaks at multiple stages in the last 15 years: 2010 with 157,202 cases, 2013 with 127,754 cases, 2016 with 101,016, and 2019 with 124,989. Previous work has approached Dengue forecasting using sensing data in China^30,31^. Similarly, other models used Dense Deep Convolutional Neural Networks on both street-level and aerial imagery combined by a Multilayer Perceptron (MLP), demonstrating that street-level images lack contextual data related to water access, which is critical for dengue prediction^32^. Oladimeji Mudele *et al*.^33^, developed a neighborhood-level forecasting framework to predict dengue using Earth Observation (EO) products and one-step ahead. Similarly, Zhichao *et al*.^34^ and Kuo *et al*.^10^ predicted Dengue using Google Earth Engine and multi-step-ahead Long Short Term Memory modeling with and without historical cases, however not considering multimodal fusion such as sociodemographic data but only environmental factors from GEE. Despite major efforts in forecasting climate-sensitive illnesses such as dengue, attaining high accuracy in model training remains a computationally intensive process, providing a significant problem in middle-income countries. We use dengue outbreak prediction to showcase how SatelliteBench embeddings contribute to accurate prediction models, even without historical Dengue data. We also demonstrate how our proposed multimodal fusion pipeline could drastically improve prediction performance.

We utilize three principal metrics: Root Mean Square Error (RMSE), Mean Absolute Error (MAE), and the coefficient of determination (R-squared). These metrics provide distinct insights into model accuracy and reliability, facilitating a thorough assessment of the model’s practical utility.

The RMSE is defined as in Eq. 1, where *y*_*i*_ are the true values, ${\widehat{y}}_{i}$ are the predicted values, and *n* is the number of observations. RMSE is particularly valuable as it penalizes larger errors more heavily by squaring the differences before averaging, making it sensitive to outliers. A lower RMSE indicates a better fit of the model to the data, signifying higher predictive accuracy:

1 $${\rm{R}}{\rm{M}}{\rm{S}}{\rm{E}}=\sqrt{\frac{1}{n}\mathop{\sum }\limits_{i=1}^{n}{({y}_{i}-{\hat{y}}_{i})}^{2}}$$

The MAE is formulated as in 2. The MAE quantifies the average magnitude of the errors in predictions, without considering their direction. MAE is robust to outliers as it does not square the residuals, providing a reliable measure of model performance across various scenarios with potential outlier data points or highly variable spreads.

2 $${\rm{MAE}}=\frac{1}{n}\mathop{\sum }\limits_{i=1}^{n}| {y}_{i}-{\widehat{y}}_{i}| $$

The R-squared is calculated using the formula 3, where $\bar{y}$ is the mean of the observed data. R-squared represents the proportion of variance in the dependent variable that is predictable from the independent variables, providing an intuitive measure of how well future outcomes are likely to be predicted by the model. A higher R-squared value indicates a more effective model with a higher proportion of variance explained.

3 $${R}^{2}=1-\frac{{\sum }_{i=1}^{n}{({y}_{i}-{\widehat{y}}_{i})}^{2}}{{\sum }_{i=1}^{n}{({y}_{i}-\bar{y})}^{2}}$$

### Time-series cross-validation for dengue prediction

**Fig. 2:**
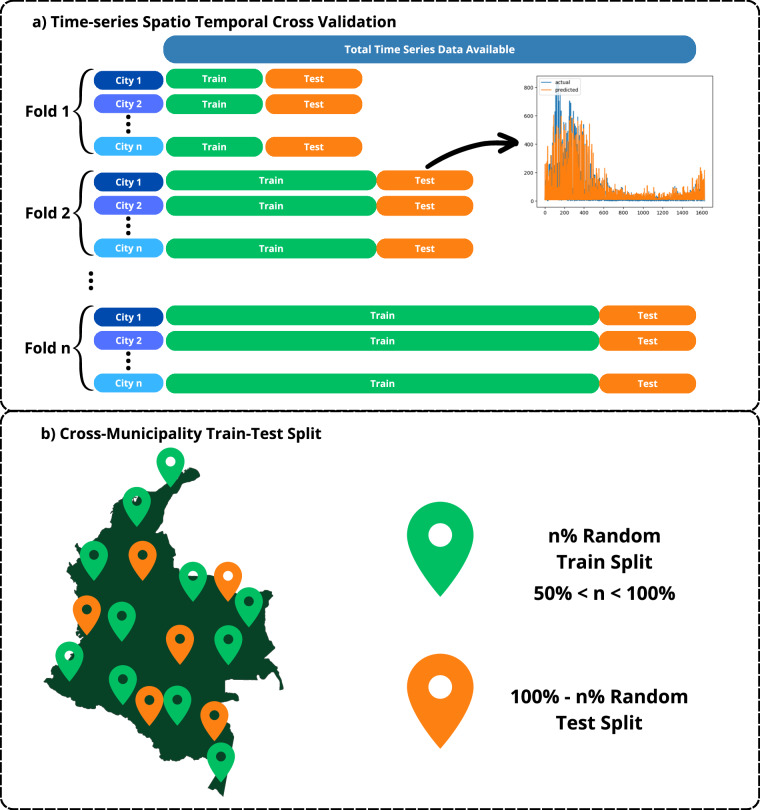
Splits used for evaluation. (**a**) shows the multi-city time-series cross-validation methodology used for temporal splits; (**b**) shows the cross-municipality split used to avoid data leakage during static splits of non-temporal data.

Utilizing the epidemiological week (Epi-week) calendar for fold assignment, we used 5 folds to construct a multi-city time-series cross-validation in our experiment (Fig. 2a). We discovered that the 5-fold strategy generated the best results over several experiments with varied splits. A sensible technique was to start training with a 6-month period and then test on the next 6-month batch, continuing this procedure, given the dataset’s data on Dengue prediction for 10 municipalities across a 2-year period (2016–2018, corresponding to 24 months). The testing data from the preceding fold was added to the following training data batch in each fold, following this pattern until the last data batch. Utilizing a single train/test evaluation split, performing cross-validation allows us to understand how the models are fitting the data because Dengue has a seasonality that occurs at least every couple of years, and that may be hard to capture from only 6 months. Given the small size of the dataset — with respect to each outbreak, using cross-validation allows to scrutinize the time-variability and its correlation with spatial patterns at a temporal level, while exploiting the capabilities of the Long-Short Term-Memory Neural Networks and combining metadata at an early stage, we are able to grasp more insightful details about the data, and allow the model to generalize outbreak prediction. On a regular problem-setting, outbreak prediction may require more years of data, so that the low-frequency component of such trends can easily be predicted. Cross-validation may allow to abstract such patterns with much ease, when combined with temporally aligned metadata. The results of this evaluation method can be seen in Table 1.

**Table 1:** Performance Across Tasks and Models for 5 Fold Time-series Cross Validation in Dengue Prediction on the Top 10 Municipalities with Most Dengue Cases.

Task	Model	RMSE	MAE	R^2^
Dengue Outbreak Prediction (Range 0–873)	M0: Fusion	68.02	28.62	0.41
	M1: M0 + Kronecker	59.98	23.93	0.63
	M2: M1 + Gated Attention	39.23	19.73	0.84
	M3: M1 + Late Dense Fusion	36.79	16.81	0.85
	M4: M3 + Mutual Information Optimization	33.87	15.38	0.88

### Multi-cross city evaluation in education and poverty

Given the fact that social indicators such as Education and Poverty are usually measured during the census, these values are considered static. For that reason, we validate the multi-modal evaluation in a multi-cross-city evaluation, where we train the models using labels from some specific cities and evaluate the model in unknown cities. The results of this evaluation method can be seen in Table 2.

**Table 2:** Performance Across Tasks and Models for Multi-cross City Evaluation in 81 municipalities using an 80%/20% Train/Test Split for Education and Poverty.

Task	Model	RMSE	MAE	R^2^
Access to Education	ME0: SVM using metadata	7.87	4.31	0.46
(Range 35–77)	ME1: SVM using metadata and Image embeddings	3.68	2.79	0.88
Poverty Mapping	MP0: SVM using metadata	6.91	5.56	0.64
(Range 5.4–50.2)	MP1: SVM using metadata and Image embeddings	7.06	4.89	0.63

### Performance and ablation studies

For dengue prediction, the overall best-performing dengue model is M4, which takes a combination of Kronecker fusion, gated attention, late dense fusion, and mutual information, capable of achieving an *R*^2^ score of 0.88 and MAE of 15.38. By doing ablation experiments, our results show that the most significant improvement factor is the dense fusion performed at later layers of the final prediction network combined with Kronecker fusion, as shown in Table 1.

In the case of access to education (Table 2) it is clear how the introduction of satellite image embeddings improves the results of the model. The use of Satellite Image embeddings in the datasets considerably increases the RMSE, MAE and *R*^2^ from 7.87, 4.31 and 0.46 to 3.68, 2.79, 0.88 respectively. In the case of Poverty (Table 2), the value of the satellite image embeddings is not clear having an improvement in the MAE from 5.56 to 4.89, but also having an *R*^2^ score of 0.64 without the embeddings and 0.63 with the embeddings. In this case, we should consider that the metadata used to build the Poverty index in Colombia is the census data, so the metadata could be used for the SVM model as a shortcut feature.

## Discussion

SatelliteBench dataset can enable a broad range of applications. While we benchmark dengue prediction, poverty mapping and access to secondary and higher education here, this is by no means restrictive. Users are encouraged to devise new applications, one of which could involve cross-referencing with extra labels and external datasets.

While prior research has explored the utility of Dengue cases and metadata in isolation, we emphasize the inherent significance of obtaining synchronized data in the context of sensing data. Our contribution lies in leveraging spatiotemporally aligned satellite image data and metadata, thereby bridging the gap between disparate information sources. Notably, our approach departs from conventional autoregressive prediction methods that heavily rely on historical dengue cases. In regions across Colombia where acquiring public health data proves cost-prohibitive, our model emerges as a crucial advancement. By exclusively employing readily available satellite images and metadata, our framework circumvents the reliance on traditional data sources, a significant boon facilitated by the tools we provide.

Moreover, our work highlights the advantages of low computational costs inherent in modeling vector-borne datasets. While conventional training with satellite images typically necessitates substantial computational resources and advanced GPUs, our methodology demonstrates remarkable efficiency. We achieved compelling predictive performances using minimalist models, such as two-layer LSTM architectures, and cost-effective GPUs. Our experiments only required the free-tier T4 GPUs offered by Google Colab, and a single complete training and evaluation cycle of our model required a mere two hours. This highlights the feasibility and accessibility of our approach, enabling more researchers to engage in effective vector-borne disease prediction with limited computational resources.

As exemplified in this paper, the geographical sample area for this study was in Colombia; nevertheless, the proposed framework is adaptable for use in any location worldwide. With respect to the selected dataset of experimentation, it has a limited time range and focuses on certain municipalities in Colombia, which may restrict its capacity to capture wider patterns. Data imbalances among labels may have an impact on predictive model performance. Dengue case data availability varies, and dengue local transmission dynamic seasonality’s thereby affecting the reliability of outbreak prediction algorithms. Even though the multimodal fusion pipeline heavily relies on satellite images and metadata, particularly for poverty and education targets, a cross-city evaluation showcases the remarkable data and model performance, yielding high results. While the models are efficient, resource constraints may hinder accessibility. The assumption that vector embeddings capture all crucial spatial and environmental information may not always hold, especially in cases lacking time-variant metadata. Despite these obstacles, satellite image vector embeddings show promise in extracting health data through cross-sectional regression tasks and cross-city evaluations. This approach has the potential for real-world decision-making applications.

### Insights about using satellite image embeddings

Vector embeddings might be a valuable tool for extracting critical information from images allowing easy and fast experimentation. The satellite images embeddings allow the extraction of the environmental features, especially in cases where analyzing raw satellite images would be impossible due to skill and computing requirements. With the proposed pipeline, we allow the extraction of vector embeddings from satellite images, and demonstrate the value of downstream tasks, demonstrating its effectiveness on climate-sensitive diseases, education, and poverty forecasting.

### Insights about the time series multimodal fusion

The time series multimodal fusion pipeline suggests an architecture that can be generalized in other time series tasks where tabular data and images are available. The use of LSTM networks allows the extraction of temporal features, while the inclusion of gated attention and Kronecker fusion allow the model to extract the most important information and mitigate the noise. The incorporation of the mutual information loss additionally allows the model to focus only on the most important features instead of paying attention to spurious features.

### Insights about predicting education

The results show the dataset’s relevance when it comes to forecasting education. The dataset’s association of 81 municipalities with education labels allows for a more comprehensive exploration of the relationships between socioeconomic characteristics and their surroundings. The results demonstrate the potential to unearth significant insights into the link between education and environmental variables by utilizing satellite image embeddings alongside metadata, thereby assisting in better informed resource allocation and policy choices.

### Insights about predicting poverty

Forecasting poverty has far-reaching ramifications. Via a cross-sectional evaluation across 81 municipalities, we demonstrate that the use of satellite images can be significantly helpful with the right metadata. Since the use of satellite image embeddings can be useful, the fusion of this data with other metadata available increases the performance of the model. In this specific instance, we illustrate the effectiveness of training on certain cities and testing on others (cross-sectional evaluation), a valuable approach in scenarios where data is scarce, and the sampling frequency surpasses weekly rates, as seen in comparison with the dengue labels.

### Insights about predicting dengue

We demonstrate accurate Dengue cases prediction using models tested through a 5-fold time-series cross-validation method, even in areas with limited prior dengue data. This is achieved by incorporating satellite image vector embeddings into our multi-modal fusion pipeline, highlighting the environment-extraction capabilities of using vector embeddings in this work. Thus, we demonstrate the dataset’s utility in addressing health issues by combining satellite images and metadata to enhance disease outbreak forecasting and support informed decision-making for public health action.

### limitations about interpretability of satellite image embeddings

While our framework demonstrates promising results in utilizing satellite image embeddings for tasks such as poverty mapping, access to education, and dengue prediction, we acknowledge the inherent challenge of interpretability associated with image embeddings. Unlike classical feature extraction methods, image embeddings lack interpretability, making it challenging to discern the specific features contributing to model predictions. Future research should prioritize improving the interpretability of image embeddings to enhance our understanding of the underlying factors influencing the models. This is crucial for ensuring trustworthiness and facilitating informed decision-making in public health applications.

### Insights about model selection

In the context of model selection, we emphasize the task-specific nature of our approach, and recognize the intricacies of each prediction task. We adapted our model choices to suit the unique characteristics of the data and objectives. The time-series multimodal fusion model proposed for dengue prediction showcases the importance of considering temporal dynamics and multimodal information. On other hand for tasks such as poverty mapping and access to education, where temporal aspects are less critical, we opted for a simpler model like SVM. We acknowledge that the choice of model should align with the inherent nature of the prediction task and caution against a one-size-fits-all approach. Our results highlight the strengths and limitations of the selected models, offering insights for future endeavors in similar domains.

### External validation

In this work we introduced a versatile multi-functional framework that aligns metadata with high-resolution satellite images spatiotemporally alongside three diverse use case scenarios across Colombia. Generalization to different temporal and spatial scales can be challenging, but our proposed framework encourages researchers to prioritize the data collection stage to create high-quality data for the ground truth. Emphasizing this step is fundamental to ensuring the robustness of the models. Initially, focus on defining the sampling frequency of the data. In this particular case, since we utilize Sentinel data, we have employed epiweeks. However, this range can be adjusted to 5 days or any scale above it. Similarly, consider the spatiogeographical scale; the data collection pipeline should encompass information for the selected resolution data that each satellite image will cover, including how temperature, precipitation, and other metadata are collected, ensuring they reflect the resolution of the given data. Once this process is finalized, proceed with training the models. To test its generalizability, we perform external validation on Brazil, as it is one of the countries with the highest incidence of dengue^35^. Brazil presents unique challenges for our framework given its different climatic conditions, populations, and less public health data availability compared to Colombia. We implemented the same next week dengue case prediction using the Brazilian dataset with a context window of 3 weeks of satellite images and metadata. Utilizing our satellite extractor tool, we gathered weekly satellite imagery for the Municipality of Rio de Janeiro from January 2016 to December 2023. The images were generated with the same parameters as the images of Colombia dataset in terms of temporal and spatial resolution, and format. This comprehensive temporal span allowed us to cover various seasonal patterns and epidemiological cycles associated with dengue transmission in Rio de Janeiro. To complement the satellite images, we extracted essential climatic metadata using MODIS (for temperature data) and CHIRPS (for precipitation data), alongside epidemiological data provided by Brazil’s public health system via the Datasus platform. It is important to mention that the ratio of metadata used for the task in Brazil was less (10%) of the total metadata used in Colombia to simulate a scenario where metadata is scarce. Results are shown in Table 3.

**Table 3:** Performance Across Models for Dengue Prediction in the External Validation Set from Brazil.

Task	Model	RMSE	MAE	R^2^
Dengue Outbreak Prediction (Range 0–2500)	M0: Fusion	211.85	137.97	0.627
	M1: M0 + Kronecker	203.40	110.01	0.655
	M2: M1 + Gated Attention	185.91	104.08	0.72
	M3: M1 + Late Dense Fusion	176.80	90.00	0.74
	M4: M3 + Mutual Information Optimization	166.49	85.59	0.77

### On model generalizability and limitations

ML robustness research such as in^36,37^ shows that training distributions *P*^*Tr*^ exhibit spurious correlations between certain features and latent classes which do not hold in the ground-truth distribution P*, causing performance drops in face of distribution shifts. Each feature vector has some useful core features (e.g. signs of humidity from satellite image) and spurious features (e.g. shadows caused by clouds). Multimodal learning has better robustness with extra information that directly correlates with outcome^38^: When predicting dengue outbreaks using satellite images only, a model might wrongly learn that all stagnant water bodies lead to dengue fever. Factors such as local policies, or mosquito species can contribute to dengue outbreaks. Fusion of metadata like local public health strategies and socioeconomic factors could help the model understand where the actual risk of dengue is, rather than making assumptions based solely on satellite imagery. Multimodal learning also benefits from paired features in some latent classes to disassociate spurious correlations in other latent classes, even if the features do not directly indicate labels^37^. Consider the task of predicting dengue outbreaks from satellite images as a scenario. A model focusing only on visible large water bodies in satellite images may not capture climatic variations like temperature that may potentially affect the mosquito life cycle. By integrating multimodal data, such as temperature or urban density, the models can learn to dissociate these spurious correlations. Denote *α* as a spurious feature’s relative magnitude to core features, *β* as strength of the model’s weight placed on the core feature, *m* as the number of latent classes, and *π* as the probability of non-label related features in the non-image data. Xue *et al*.^36^ proves that multimodal models can only achieve theoretical maximum accuracy on P* if $\pi  > \frac{{(1+\beta )}^{2}\alpha -1+\beta }{{(1-\beta )}^{2}{\beta }^{2}(m-1)}$ Our mutual information loss further helps reduce the redundancy between shared modality information and modality-specific information and disentangles the spurious and useful features for prediction. Additionally, dense fusion allows for more complete interactions between features across modalities, thus making the model stronger to out-of-distribution shifts.

However, as discussed above, the robustness of multimodal learning really depends on how much auxiliary metadata is collected. Lack of sufficient metadata may hurt the performance, as is shown in the performance drop of our generalization test in Brazil, where we collected around 10% of the metadata compared with the original dataset. Our findings underscore the challenges posed by spurious correlations within training distributions, which can significantly affect the model’s performance when exposed to real-world, heterogeneous datasets.

It is also important to keep in mind that the model has not been stressed in other tasks and with other scale variations in different periods. But it is encouraged if there is enough data to perform such multiple-step ahead predictions at day or weekly scales. Taking this into account, the use of good practices such as data normalization and regularization techniques is recommended to obtain a good model performance.

To improve the generalizability of our models, future work should include testing the models across a broader range of environments and use cases. This could involve extending the model applications to other regions with different climatic and socio-economic characteristics, thereby testing the models’ resilience and adaptability.

### On framework generalizability and limitations

Although achieving a 0.77 *R*^2^ score on the Brazilian external validation, our model experiences performance drop as compared to Colombia, indicating nuances of applying the framework across different regions. The generalizability issue could potentially be mitigated by engaging more deeply with local knowledge and resources, as the amount of metadata available is considerably less in Brazil compared to Colombia. While our framework can extract lightweight satellite imagery embeddings from diverse locations and time, the collection of metadata heavily relies on public agencies operating at the municipal, regional, and national levels. Hence, our proposed framework encourages researchers to consider this as a baseline or reference point and analyze the real-life challenges when integrating data from both public and private institutions for their desired regions of interest. However, it is worth noting that our satellite image plus metadata prediction framework is still a much cheaper alternative to traditional prediction relying on historical epidemiological data, which is often unavailable in real-life scenarios.

Our research demonstrates that while our satellite image extraction methodology and the mutual information fusion model provide robust foundational tools, there is no solution that can be blindly applied across all global institutions^39,40^. The models developed using satellite imagery often face limitations in generalizability across different geographic regions due to regional variability in environmental, socio-economic, and health conditions.

Additionally challenges are presented due to inconsistencies and gaps in metadata collection can significantly hinder the standardization and effectiveness of satellite-based predictive models. The scarcity of comprehensive metadata, such as detailed local health statistics or environmental data, limits the depth and accuracy of analyses, thereby constraining the potential impact of such models on public health decision-making.

To overcome the challenges associated with verifying ground truth data in diverse regions, we propose a multifaceted approach that includes the establishment of local partnerships for data collection, the adoption of advanced data fusion techniques, and the utilization of high-resolution satellite data complemented by robust geospatial analysis tools. Furthermore, implementing open data appraches^41–43^, and synthetic data generation^44,45^ can provide additional support where data gaps exist. We also recommend the development of standardized data verification protocols that can be adapted for use across different geographical settings, ensuring the consistency and reliability of data used to validate and recalibrate our model. Transparent reporting and collaborative data sharing should be encouraged to enhance model verification processes and foster a community of practice that supports continuous improvement and adaptation of the model.

The future scenarios involving climatic changes pose additional uncertainties for predictive frameworks relying on satellite imagery. Shifts in climate patterns can alter the physical landscape, affecting the accuracy of models that predict phenomena such as vector-borne diseases, water-borne illnesses, or agricultural output. Changes in vegetation cover, water bodies, and urban expansion, all detectable via satellite, require continuous monitoring and model recalibration to ensure that predictions remain accurate under changing climatic conditions. Moreover, the inclusion of external factors such as global pandemics can drastically alter the expected behavior of public health outcomes, thus impacting the predictions made by satellite-based models. For instance, the onset of a pandemic could change human mobility patterns, land usage, and even local environmental conditions, all of which are typically captured indirectly through satellite imagery. These shifts necessitate models to be flexible and adaptive, incorporating real-time data feeds and regularly updated training cycles to remain relevant and accurate.

To mitigate these challenges, it is imperative to design frameworks that are not only robust but also inherently adaptable to changing data characteristics. This involves integrating hybrid modeling approaches that combine static historical data with streams of current data. Employing advanced machine learning techniques such as transfer learning and ensemble models can also aid in adjusting to new patterns as they emerge. Furthermore, developing a systematic approach to incorporate auxiliary data sources—such as local weather stations, IoT sensors, and crowd-sourced information—can enhance the model’s sensitivity to real-time changes. Continuously updating the training sets to include recent data and reevaluating the model assumptions are essential strategies to handle uncertainties in data caused by pandemics or climatic changes. This adaptive learning approach ensures that the models evolve in response to new data, reducing the risk of obsolescence. Collaborations with climatologists and epidemiologists can also provide predictive models with forward-looking data that anticipate major environmental or health shifts, thereby pre-emptively adjusting the models’ parameters.

We encourage the research community to leverage their local expertise^46^, identifying unique problems and variables pertinent to their specific contexts^46^. This collaborative approach not only ensures the relevance and effectiveness of deployed models but also fosters a more inclusive and informed community around the use of satellite data for public health.

### Technical challenges

In leveraging satellite imagery for public health predictions, several technical challenges must be acknowledged. The variability in image quality, influenced by factors such as cloud cover, atmospheric conditions, and sensor limitations, poses significant challenges^47^. These issues can lead to inconsistencies in the data, affecting the accuracy and reliability of subsequent analyses. Furthermore, the temporal resolution of satellite data, determined by the frequency of satellite over specific regions, may not always align with the temporal needs for real-time data analysis. In this study, although we included a framework for the extraction of satellite images spatially and temporally, this framework is limited to the maximum temporal resolution provided by Sentinel 2, which is 5 days^48^. A similar problem occurs in terms of spatial resolution, where the maximum spatial resolution obtained is 10 meters per pixel, limiting applications to applications with a temporality greater than 5 days and that do not require a spatial resolution of less than 10 meters such as people^49^ or vehicle detection^50^.

Additionally,challenges in terms of computational resources and expertise are posed. The processing and analysis of large volumes of high-resolution satellite imagery demand significant computational resources, which pose a challenge in resource-constrained settings. Additionally, adopting advanced satellite-based predictive models necessitates substantial technical expertise, which may not be uniformly available across all public health environments. The effective implementation of these technologies requires targeted training programs and capacity-building initiatives to equip health professionals with the necessary skills to leverage satellite data fully.

### The spatial statistic trinity (SST) framework

The Spatial Statistic Trinity (SST) framework^51^, facilitates the articulation of sampling decisions by considering various factors related to the entire population of interest, such as the geographic location, demographic features, historical dengue incidence rates, and other relevant variables. This is a framework that models a balance between the design based approach and model base approach by taking into account the spatial autocorrelation (SAC)^52^ and spatial stratified heterogeneity (SSH)^53^. However, in our specific case, the selection of cities for sampling the dengue cases was driven by epidemiologists experts on the topic of dengue who told us which cities would be of greatest interest given the prevalence of dengue. We prioritized cities based on their history and current status of dengue proliferation, ensuring that our sample captured a diverse range of scenarios and challenges related to dengue prediction. By focusing on cities with significant dengue incidence rates, we aimed to develop and validate our ML model in contexts where the disease burden is most acute, thus enhancing the relevance and applicability of our findings to similar settings. In the other use cases the sampling was more extents to cover a broader range of geographic and demographic conditions covering 81 municipalities.

To extend the use of the SST to our dataset, and understand the distribution of SAC and SHH present in our population, we performed a SAC analysis using Moran’s I^54^ and Geary’s C. Moran’s I was defined in Eq. 4 as:

4 $$I=\frac{n}{W}\frac{{\sum }_{i=1}^{n}\,{\sum }_{j=1}^{n}{w}_{ij}({x}_{i}-\bar{x})({x}_{j}-\bar{x})}{{\sum }_{i=1}^{n}{({x}_{i}-\bar{x})}^{2}}$$

Where:

*I*: Moran’s I index

*n*: Number of observations

*W*: Sum of the weights in the spatial weights matrix

*x*_*i*_*, x*_*j*_: Values of the variable at locations i and j respectively

$\bar{x}$: Mean of the variable

*w*_*ij*_: Spatial weight between locations *i* and *j*

Geary’s C was defined as in the Eq. 5:

5 $$C=\frac{(n-1)}{2W}\frac{{\sum }_{i=1}^{n}{{\sum }_{j=1}^{n}{w}_{ij}({x}_{i}-{x}_{j})}^{2}}{{\sum }_{i=1}^{n}{({x}_{i}-\bar{x})}^{2}}$$

Where:

*C*: Geary’s C index

*n*: Number of observations

*W*: Sum of the weights in the spatial weights matrix

*x*_*i*_*, x*_*j*_: Values of the variable at locations *i* and *j* respectively

$\bar{x}$: Mean of the variable

*w*_*ij*_: Spatial weight between locations *i* and *j*

Moran’s I values close to 1 suggest high positive correlation (similar values clustered together), while values close to −1 suggest high negative correlation (dissimilar values clustered together), and values near 0 suggest no spatial correlation. Geary’s C on the other hand, shows strong autocorrelation when its values are near zero, 1 when there is any spatial autocorrelation and 2 when it has a negative spatial autocorrelation. The spatial weight matrix, denoted as *W*, is crucial for defining the spatial relationship among observations since influences how the spatial autocorrelation of each observation with respect to its neighbors is computed.

To construct *W*, we consider a binary distance-based method where two observations are considered neighbors if they are within a predefined threshold distance. Formally, the weights matrix *W* is defined as follows:

6 $${w}_{ij}=\left(\begin{array}{cc}1 & {\rm{if}}\;{d}_{ij}\le \theta \\ 0 & {\rm{if}}\;{d}_{ij} > \theta \end{array}\right.$$

Where:

*w*_*ij*_ is the element of the matrix *W* representing the spatial weight between observation *i* and observation *j*

*d*_*ij*_ is the geographical distance between observation *i* and observation *j*

*θ* is the distance threshold determining the neighborhood.

The matrix is symmetric assuming that the distance measure used is symmetric, and *w*_*ii*_ = 0 for all *i*, ensuring no self-influence in the calculation of spatial autocorrelation indices.

The overall sum *W* in Eqs. 4 and 5, which is used to normalize the measures, is simply the sum of all weights in **W**, given by:

7 $$W={\sum }_{i=1}^{n}{\sum }_{j=1}^{n}{w}_{ij}$$

experiments were executed inspired on^55^. Based on the results presented in Table 4, it is evident that certain metadata variables exhibit notable spatial autocorrelation within the overall superpopulation dataset. Notably, latitude and longitude demonstrate the highest levels of spatial autocorrelation, which is reasonable given that the data is confined within Colombia. Additionally, building stratification and select population demographics, such as population density among individuals aged 5–14 and those above 30 years old, exhibit significant spatial autocorrelation. This trend is consistent with environmental factors observed from 2016 to 2018, including precipitation and temperature.

**Table 4:** Moran’s I, Geary’s C top 20 most autocorrelated metrics per population variable.

Variable	Moran’s I	Geary’s C
Latitude	0.947	0.018
Longitude	0.908	0.04
Building stratification 1 (%)	0.591	0.422
Age 5–14 (%)	0.553	0.37
Age > 30 (%)	0.542	0.414
2017_precip	0.535	0.388
2016_precip	0.525	0.397
2018_precip	0.52	0.406
2017_temp	0.503	0.506
2016_temp	0.503	0.507
2018_temp	0.5	0.509
Building stratification 2 (%)	0.496	0.511
Age 0–4 (%)	0.462	0.485
Employed population (%)	0.412	0.556
Indian Population (%)	0.41	0.513
Afrocolombian Population (%)	0.384	0.551
Households without internet access (%)	0.348	0.662
Age 15–29 (%)	0.345	0.652
Households without water access (%)	0.298	0.647

**Fig. 3:**
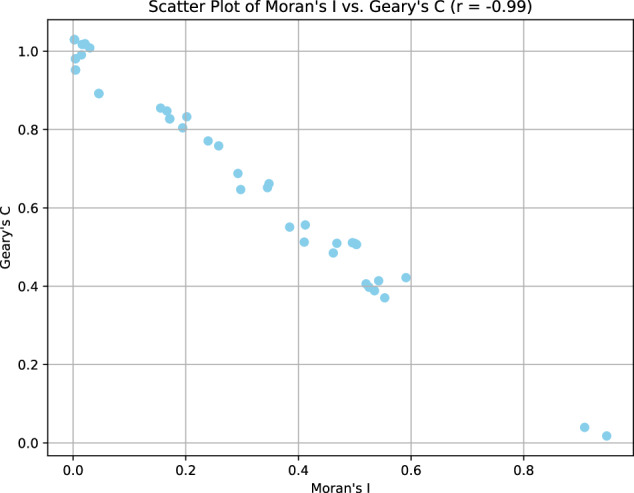
Scatter plot for all municipalities analysis with Moran’s i vs Geary’s C.

Moreover, various population density attributes demonstrate interrelationships, such as employment rates, the presence of indigenous and Afro-Colombian populations, households lacking internet access, and those with access to water resources. Additionally, as depicted in Fig. 3, only two variables exhibit pronounced autocorrelation: latitude and longitude. Conversely, other variables display moderate to low spatial autocorrelation.

For SSH we used the method proposed by Wang, *et al*. pd-value^56^, and renamed q-statistic in^15^. The q-statistics are used in multiple works as a way to measure the spatial heterogeneity of certain population under certain demographic or geographic conditions^53,57,58^, and can be used as a way to determine if the sampling method should be stratified or can be random^15^.

To calculate SSH, k-means clustering was employed to define strata based on variables of interest—dengue cases, poverty index, and access to school—alongside geographic coordinates (latitude and longitude). Prior to clustering, min-max normalization was applied to each variable to ensure equal contribution to the analysis, scaling them to a uniform range of [0,1]. This normalization is crucial given the disparate scales of the input data, and is defined mathematically as:

8 $$x{\prime} =\frac{x-\mathrm{min}(x)}{\mathrm{max}(x)-\mathrm{min}(x)}$$

where *x* represents the original value, and *x*′ the normalized value.

The k-means algorithm partitions *n* observations into *k* clusters, where each cluster is defined to minimize the within-cluster sum of squares. The objective function for k-means is given by:

9 $$\mathop{\arg \,\min }\limits_{{\bf{S}}}\mathop{\sum }\limits_{i=1}^{k}{\sum _{{\bf{x}}\in {S}_{i}}\left\Vert {\bf{x}}-{\mu }_{i}\right\Vert }^{2}$$

with *μ*_*i*_ representing the mean of points in *S*_*i*_.

Following the determination of strata via k-means clustering, the q-statistic is calculated to quantify the degree of SSH. The q-statistic is defined in^53^ as:

10 $$q=1-\frac{{\rm{SSW}}}{{\rm{SST}}}$$

where SSW is the within-stratum sum of squares, and SST is the total sum of squares across the dataset, with *h* representing the strata clusters:

11 $${\rm{SSW}}=\mathop{\sum }\limits_{h=1}^{k}{\sum _{i\in {S}_{h}}({y}_{i}-{\bar{y}}_{h})}^{2}$$

12 $${\rm{SST}}=\mathop{\sum }\limits_{i=1}^{n}{({y}_{i}-\bar{y})}^{2}$$

The q-statistic measures the proportion of the variance in the dataset explained by between-stratum differences rather than within-stratum variations. A higher q-value indicates a greater degree of spatial stratified heterogeneity, suggesting that significant variation across the dataset is due to differences between the defined strata.

The analysis of the q-statistics from various variables (Table 5) demonstrates moderate spatial stratified heterogeneity in the impacts of demographic and environmental factors on Dengue, poverty, school access and population. In the table relations across the q-statistic values of dengue with respect to population, the value decreases over time, showing a the complexity of the analysis due to temporal challenges. Additionally the heterogeneity of dengue with respect to socioeconomic, climatic, and demographic variables can be also seen for variables such as poverty, building stratification or temperature.

**Table 5:** Q-statistics for different strata variables using clusters based on k-means with respect to latitude and longitude and each variable, including population.

Variable	Q-statistic			
	Dengue	Access to School	Poverty	Population
2016 Dengue	0.943784	0.051018	0.008522	0.940676
2017 Dengue	0.825310	0.050365	0.009121	0.780181
2018 Dengue	0.705498	0.074015	0.066382	0.162896
Building Stratification 1 (%)	0.476898	0.503625	0.515955	0.483693
Poverty	0.426974	0.497315	0.874162	0.414546
Building Stratification 2 (%)	0.359510	0.427901	0.463524	0.351874
2017 Temperature	0.347884	0.346788	0.437341	0.328342
2016 Temperature	0.347530	0.346233	0.435820	0.327865
2018 Temperature	0.344033	0.343055	0.433127	0.324044
Age > 30 (%)	0.316556	0.279309	0.478305	0.290825
Households without Internet Access (%)	0.298172	0.472694	0.355420	0.292282
Age 5–14 (%)	0.286646	0.358289	0.495638	0.273451
Age 0–4 (%)	0.281561	0.240912	0.443480	0.263498
2017 Precipitation	0.260314	0.201123	0.499917	0.258510
2018 Precipitation	0.250707	0.198107	0.458185	0.248624
Age 15–29 (%)	0.250467	0.246600	0.273096	0.227328
2016 Precipitation	0.233115	0.184782	0.486835	0.223732
Number of Hospitals per Km^2^	0.228446	0.111497	0.039515	0.142067
Employed Population (%)	0.198708	0.273717	0.334767	0.195146
People who Cannot Read or Write (%)	0.189148	0.495266	0.439594	0.185355

SatelliteBench is inherently governed by SST, showing a high influence of the SAC and SSH to different variables under different spatial and temporal scales. The SST and the selected cities suggests a systematic approach to city sampling, to potentially mitigate biases in model training. This correlation between temperature and dengue proliferation offers a methodical way to select cities, ensuring a better selection bias for training models within the SatelliteBench framework. It’s noteworthy that while low SAC (Spatial Autocorrelation) may allow random sampling, it doesn’t imply that city selection should be solely based on randomness. Instead, our approach considers the interdependence of cities, acknowledging that their selection should be guided by factors such as dengue prevalence, data availability, and expertise. The selection of the cities shared in Physionet was driven by the richness and diversity of dengue data available, further reinforcing the suitability of the approach.

### Future directions

This work marks an important step in public health research, as we show that prediction of important outcome variables like dengue cases does not necessarily have to rely on expensive historical epidemiology data collection. It’s worth acknowledging the considerable challenge posed by the labor-intensive nature of collecting and aligning metadata at the municipal level when it comes to generalization. Comprehensive metadata collection will still be crucial for ensuring top model performance across populations. In the future, it is essential to streamline and potentially automate public metadata acquisition with technologies like large language models, and strengthen collaborations with local entities to facilitate attributes acquisition, to enhance the scalability and efficiency of this framework across diverse geographic contexts. We will also expand the usability of our framework by including features like image resolution upscale, and diffusion-models-based cloud and shadow removal. By sharing tools, methodologies, and datasets, we aim to empower researchers and public health practitioners worldwide to adapt and apply these approaches in their local contexts.

## Methods

The SatelliteBench dataset^26^ was created using a multimodal data extraction framework, as shown in Fig. 1, where satellite images are spatiotemporally matched with their related metadata. In the first stage, the dataset is parametrized in this framework’s three stages by entering only the desired geographic coordinates, starting date, and ending date. In the second stage, black images are corrected using our image selective method. Finally, in the third stage, we demonstrate the differential encryption-based extraction and qualitative assessment of satellite images. We show how the data aligns with the associated metadata when access to public sources is available on a temporal basis. These steps are explained in more detail in the subsequent sections.

### Satellite imagery image extraction

We extracted 12,636 satellite images per Epi-week using the Sentinel-2-L1C and Sentinel-2-L1A satellites. Sentinel-2 satellites have a 5-day revisit time between them and cover the globe at a spatial and temporal level, capturing stationary and environmental changes. Since the resolution of Sentinel-2 varies between 10 m and 60 m depending on the spectral band of the satellite, we upsampled all bands to a 10 m resolution using nearest neighbor interpolation from the Sentinel Hub API. The used bands were B02 (Blue, 492.4 nm), B03 (Green, 559.8 nm (S2A)), B04 (Red, 664.6 nm), B08 (NIR, 832.8 nm) with 10 meters spatial resolution; B05 (Vegetation red edge, 704.1 nm), B06 (Vegetation red edge, 740.5 nm), B07 (Vegetation red edge, 782.8 nm), B11 (SWIR, 1613.7 nm), B12 (SWIR, 2202.4 nm) with 20 meters spatial resolution; B01 (Coastal aerosol, 442.7 nm), B09 (Water vapor, 945.1 nm), B10 (SWIR — Cirrus, 1373.5 nm) with 60 m spatial resolution.

In stage 1, we designed a customized framework to download spatiotemporal aligned satellite images based on the Epi-week using the Sentinel Hub API. The framework allows users to specify latitude and longitude coordinates, start and end dates of the study, municipality code, image length, image format, and spatial resolution, as well as Google Cloud Platform (GCP) and Sentinel Hub credentials^59^ 41.

In the second stage, we downloaded the best possible image from each Epi-week based on the least cloud coverage (leastCC) mosaicking order algorithm provided by the Sentinel Hub API. This method selects pixels with the least cloud coverage metadata within an Epi-week interval. Despite applying the leastCC mosaicking order algorithm, there were still instances of repeated images per Epi-week. This is because local environmental and satellite measurement conditions can lead to several cloud-occluded, and noisy images being captured over months. To obtain high-quality images with the least cloud occlusion and noise artifacts per Epi-week, we recursively removed black images, where the sum of pixels equals zero. This was done by moving forward in time until we obtained images with non-zero pixel values for a given week. This framework was dockerized to promote reproducibility and scalability in future application deployments.

In the third stage, we implemented a hash analysis to assess the spatiotemporal variation of the satellite images by indicating the relative frequency of the duplicated images. The goal of hash encryption is to create a unique fingerprint for each image per Epi-week so that even the change of station or cloud interference on a single pixel creates a different hash and thus evaluates neighboring spatiotemporal images based on the Epi-week calendar. We used the difference hash (dhash), because it evaluates neighboring pixels by tracking the gradients. This algorithm provides scale, brightness, and contrast invariance, and ensures that features remain constant in our multi-spectral dataset. We defined the hash function as *F*, which converts a 2D domain *I*, to a fixed small range output *y* = *F*(*I*). The output y does not reveal information about the input, and it is hard to find collisions^60^. Thus, for a collection *C*, we use a hash function *F* to map a unique fingerprint y to each element of that collection.

### Metadata extraction

Metadata corresponding to each city was extracted using the municipality code of each city, which is a unique numeric identifier of a city in Colombia. The data extracted was both static and ongoing, with different time resolutions of weeks or months. The static data was extracted to include some variables that represent the social determinants of health (SDOH), like the indexes of poverty, access to school, and access to water, among other variables, and sociodemographic variables like distribution of gender, distribution of age, and population, among others. The sociodemographic variables were extracted from the National Administrative Department of Statistics of Colombia (DANE) using the data from the last census in 2018^61^.

The dynamic data was used to represent the epidemiological and climatic metadata. The number of cases of Dengue per Epi-week was extracted from the Colombian Public Health Surveillance System (SIVIGILA) website^62^. Dengue was chosen as the epidemiological case study due to its susceptibility to climate change, with factors like temperature, rainfall, and relative humidity influencing transmission. Additionally, landscape features such as water bodies, human settlements, and vegetation have been linked to the disease. Climatic variables like temperature and precipitation were extracted monthly for each city using worldclim^63^ for the 81 municipalities.

Finally, weekly temperature and precipitation in the top ten municipalities with the most dengue cases were extracted for the generation of baseline models. These data were extracted using the Google Earth Engine. Daily temperature was extracted for the whole municipality using MODIS^64^ and precipitation was extracted using CHIRPS^65^. The daily data per municipality was grouped by taking the values inside the coordinates of the region of interest (ROI) and taking the mean of those values daily. The daily values were then grouped by Epi-week, using the mean for the temperature, and summing for the precipitation.

### Spatial-temporally aligned image data, social determinants, and climate-sensitive diseases

Our extensive benchmark dataset covers 81 municipalities in Colombia from 2016 to 2018 and is primarily focused on the relationship between social variables and climate-sensitive diseases^26^26. This dataset creates a vital connection between satellite images and metadata, whose alignment is brought together by the JSON file structure, which provides crucial fields like Municipality Code (geographic reference), Epi-week (temporal reference), Image Path (image linkage), Static Data (socioeconomic context, including education and poverty), Multi-Class Labels (case status for dengue), and Continuous Data (cases, climatic data). An effective platform for examining the complex interactions between social factors, climate-sensitive diseases, and local dynamics across both the ten cities and the 81 municipalities is provided by this standardized format, which optimizes data organization, scalability, and flexibility.

### Vector embedding generation

Processing and analyzing satellite images require high-computational resources. Therefore, using vector embeddings of these satellite images provides a fast-efficient method for extracting, analyzing, and seamlessly integrating complex spatial and environmental data. In this part, we explain the process used to produce vector embeddings using our proposed framework.

We employed a ResNet-based Variational Autoencoder (VAE)^66^ through self-supervised learning to generate versatile embeddings for satellite images. Self-supervised learning involves training the model without explicit labels, allowing it to autonomously learn representations. This approach is particularly advantageous for satellite image analysis, overcoming challenges associated with limited labeled data. By using self-supervised learning, our framework ensures the adaptability and generalizability of vector embeddings across diverse tasks without the need for task-specific labels or retraining. This strategy aligns with our goal of bridging data gaps in economically unfeasible regions and enhances the efficiency of our framework. Self-supervised learning contributes to the scalability and accessibility of our approach, making it well-suited for resource-constrained environments.

Vector embeddings are a method to represent high-dimensional data (such as images) in a lower-dimensional space, preserving essential information while reducing dimensionality^67^. For an image *X* with dimensions $X\in {{\mathbb{R}}}^{h\times w\times c}$ (height, width, and channels), the goal is to transform *X* into a vector representation ${\rm{Z}}\in {{\mathbb{R}}}^{d}$ where $d\ll h\times w\times c$. This transformation can be defined as:

13 $$Z=f(X)$$

Where:

- $f:{{\mathbb{R}}}^{h\times w\times c}\to {{\mathbb{R}}}^{d}$ is a function that maps the high-dimensional image data to a lower-dimensional embedding space.

**Fig. 4:**
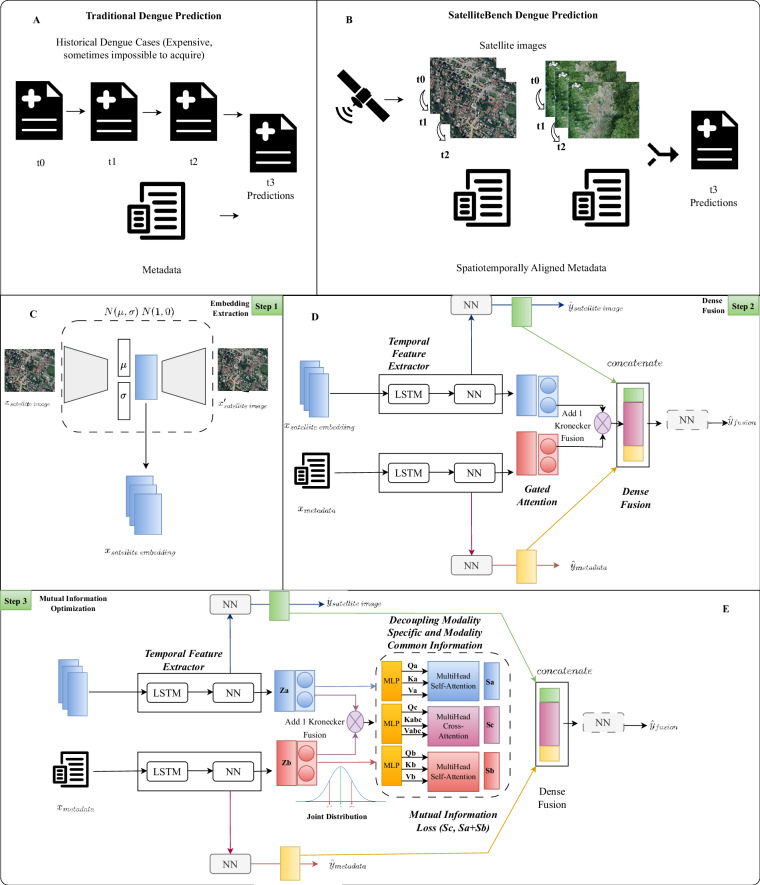
Time-series Embedding Fusion Pipeline. (**A**) Traditional Dengue Cases Prediction. Traditional dengue prediction is performed using the historical data as part of an auto-regressive model, using the cases in the previous weeks to predict future weeks. (**B**) SatelliteBench Dengue Prediction. Our proposed approach uses a time-series of satellite images to predict the number of cases avoiding the need of historical epidemiological data. (**C**) Embedding Extraction. Proposed pipeline to extract vector embeddings from the satellite images to generate cheaper and easier to implement models. (**D**) Time-series Fusion Model. The proposed model uses a sequence of embeddings of satellite images together with other information like temperature, precipitation and socio-demographic data to predict dengue cases. (**E**) Mutual Information Optimization. The Model utilizes a disentangled transformer to decouple modality-specific and modality-common information and reduce the information redundancy.

A VAE approach was applied in this research to develop multi-proposal embeddings via self-supervised learning. As shown in Fig. 4c - Step 1, the embeddings were created by training an asymmetric variational autoencoder designed expressly to recreate the satellite images. After comparing several encoder designs, embeddings produced by ResNet50 V2 were finally selected to be used in downstream tasks.

In VAEs, we begin by transforming an input image $X\in {{\mathbb{R}}}^{h\times w\times c}$ into a lower-dimensional latent vector $z\in {{\mathbb{R}}}^{d}$, where $d\ll h\times w\times c$. This transformation effectively compresses the image data while preserving its essential characteristics:

14 $$z={f}_{\phi }(X)$$

Here, *f*_*ϕ*_ represents the encoder part of the VAE, parameterized by *ϕ*, which maps the high-dimensional input data to a lower-dimensional latent representation. The encoder outputs parameters *μ* and *σ*^2^, defining a Gaussian distribution from which the latent vector *z* is sampled^66^ as defined in 16 where $\varepsilon  \sim {\mathcal{N}}(0,I)$:

15 $$\mu ,\log \,{\sigma }^{2}={{\rm{Encoder}}}_{\phi }(X)$$

16 $$z=\mu +\sigma \cdot \varepsilon $$

The training of the VAE invelves the described encoder ${q}_{\phi }(z| X)$, that parameterizes a latent distribution; and a decoder ${p}_{\theta }(X| z)$, parameterized by *θ*, that reconstructs the input *X* from the latent code *z*:

$$\widehat{X}={{\rm{Decoder}}}_{\theta }\,(z)$$

The VAE model was trained using the RGB bands of the images of the 81 municipalities to regenerate the images. The images were cropped in the center to match with the input and output shape of the model, which was 224 × 224 × 3. The model was trained during 55 epochs using an Adam optimizer with a learning rate of 0.001 and a batch size of 16.

Regarding the design of the asymmetric variational autoencoder, we used two dense layers, each with 1024 neurons, to generate the mean and variance of a normal distribution. This normal distribution samples to a fixed latent space of 1024, which was used to generate the embeddings of the satellite images. The decoder architecture was defined as 3 blocks each block composed by a 2D deconvolution, a batch normalization and a leaky relu. The number of filters in the deconvolution of each block were 128, 64 and 32. A final deconvolution was applied to sample to the original input shape 224 × 224 × s3.

The training objective of a VAE is encapsulated in the Evidence Lower Bound (ELBO)17^66^, which integrates reconstruction quality with a regularization term derived from the Kullback-Leibler divergence:

17 $${\mathscr{L}}(\theta ,\phi ;X)={{\mathbb{E}}}_{{q}_{\phi }(z| X)}[\log \,{p}_{\theta }(X| z)]-\beta \cdot KL({q}_{\phi }(z| X)| | p(z))$$

The first term of the ELBO aims to maximize the expected log-likelihood of the data, focusing on how well the reconstructed data $\widehat{X}$ matches the original input *X*. This term evaluates the reconstruction quality.

The second term, the KL divergence, ensures that the distribution of the latent vectors as modeled by the encoder remains close to a prior distribution, typically a standard normal distribution ${\mathscr{N}}(0,I)$.

The KL loss can be seen in the Eq. 18. This loss was used to direct the embedding representation towards a normal distribution, ensuring that the produced embeddings follow a normal distribution with a mean of 0 and a standard deviation of 1, avoiding sparse embeddings. In this case the values of *μ* and *σ*^2^ represented by two dense layers in the architecture, and *d* is the number of neurons, in this case 1024.

18 $$KL(\mu ,{\sigma }^{2})=-\frac{1}{2d}\mathop{\sum }\limits_{i=1}^{d}(1+{\rm{l}}{\rm{o}}{\rm{g}}\,({\sigma }_{i}^{2})-{\mu }_{i}^{2}-{\sigma }_{i}^{2})$$

The RL can be seen in the Eq. 19. A Mean Squared Error (MSE) was used as RL to evaluate the model’s capacity to accurately recreate the original image using the distribution in the embedding space. In this case y is the original image, y is the generated image, and n is the batch size.

19 $$RL(\widehat{y},y)=\frac{1}{n}\mathop{\sum }\limits_{i=1}^{n}{\left\Vert {y}_{i}-{\widehat{y}}_{i}\right\Vert }^{2}$$

### Experimental setup for use case scenarios

To validate the proposed framework and show the value of the satellite image embeddings, we used the dataset generated in 3 scenarios of common use cases in public health, which are the prediction of dengue cases, the prediction of poverty and access to schooling. Because dengue is a disease highly sensitive to climate change, the prediction of dengue cases must be done weekly and considering the previous weeks. Due to the temporary nature of dengue, a time-series approach was used in this scenario.

Although our methodology primarily relies on the generation of vector embeddings to capture relevant spatial and environmental information; we recognize inherent challenges in image quality. Arising from factors such as clouds, shadows, and missing can be still present in the dataset even with the algorithms applied during the extraction. To address these limitations and ensure the robustness of our models, we propose a data fusion approach that integrates additional information from non-image sources, as can be seen in Fig. 4, step 2.

In cases where image or embedding quality may be sub-optimal, our data fusion strategy allows for the incorporation of external data, ensuring continuity in the information flow. This approach involves the integration of metadata encompassing sociodemographic variables, poverty indices, and climatic data, obtained from non-image sources. By combining these diverse data sources, our framework aims to provide a more comprehensive context for the satellite images, enhancing the overall performance and reliability of our predictive models.

### Time-series scenario for dengue cases prediction

We first trained baseline models that only use metadata and satellite image embeddings, then a novel fusion model (shown in Fig. 4d - Step 2) and a mutual information optimization (shown in Fig. 4e - Step 3) that addresses the challenging task of multimodal time series prediction for dengue outbreaks using satellite images and metadata. Our method uses time-series metadata consisting of temperature, precipitation, and static data consisting of socio-economic and socio-demographic factors as inputs for the metadata model, and VAE with Resnet50V2 encoder extracted satellite image embeddings as inputs for the embeddings model. All experiments were conducted for a time segment of 3 given the life cycle of the mosquito^33,68,69^, epoch of 50, and batch size of 16.

### Metadata only & embedding only models (temporal feature extractors)

We considered a 2-layer long short-term memory network (LSTM) where the first layer consists of 1000 neurons and the second layer consists of 500 neurons. The output from the second layer was passed to two dense layers with 256 neurons and 128 neurons each. ReLU activation was used as part of the dense layers. We applied batch normalization before each dense layer. The final dense layer outputs were passed to a 1-neuron layer with linear activation. This LSTM-based encoder was used to train two separate models using only metadata and only satellite image embeddings respectively.

#### Multimodal fusion

To generate unimodal representations, we took the second layer of dense embedding from each extractor, which gives us the image and metadata vectors. Huang *et al*.^70^ introduced Early, Late and Joint Fusion by categorizing the methodologies into those three distinct groups: early fusion (which involves combining the raw inputs from each modality as input of a model), joint fusion (where learned intermediate features from each modality are integrated in a model), and late fusion (entailing the combination of predicted probabilities derived from each modality). We tried out early and late-joint fusion: During the early fusion model training, the image embedding encoder and metadata encoders were first trained and then kept frozen when the parameters of the fusion model were being optimized; During the late-joint fusion training, the entire pipeline was optimized together.

#### Gated attention & Kronecker fusion

Inspired by Chen *et al*.^71^, after the unimodal temporal feature vectors were learned separately, we applied a Kronecker product fusion controlled by gated attention. Before the fusion, we append one to metadata and image embedding to preserve the unimodal information. Then, we applied the Kronecker product to multiply every neuron to generate multimodal representation, which captures all cross-modality interaction between metadata and images. A gated attention module was applied to both the image and metadata features before fusion to control the expressivity of each modality and prevent noisy features. To prevent potential collinearity from one modality from dominating the other, we computed the element-wise product of the unimodal and attention scores. The output then went through an additional fully connected fusion layer of size 512 to generate a fused embedding.

#### Late dense fusion

Inspired by the dense fusion method^72^, we additionally learned a deeper representation of the image and metadata features with two separate fully connected networks. We then concatenated a dense representation that aggregates both the two deeper feature vectors and the fused embedding from the previous step. Note that, different from^72^, during our early fusion type of experiments, our LSTM + NN extractors for both modalities were still kept frozen and only the additional Fully Connected Neural Networks (FCNN) were optimized. Following our setup for uni-modality learning, we appended a FCNN prediction module after the dense fusion embeddings to get the final prediction. We experimented concatenating the deeper vectors with the Kronecker fusion vector at different layers of the final prediction module and found that it works better when the dense fusion takes place in deeper (later) layers of the prediction network.

#### Mutual information optimization

To efficiently fuse the overlapping yet critical information that both modalities share, known as the “inter-modal redundancy” challenge^73^, and reduce spurious features, we additionally approach the problem from an information theory angle. Each modality has its own information to noise ratio and may have duplicate or spurious features that impedes efficient learning. To better regularize the information redundancy, we utilize a disentangled transformer architecture^73^ that decouple the multimodal data into modality-common features *S*_*c*_ and modality-specific features *S*_*a*_, *S*_*b*_, where *a* denotes image and *b* metadata. Features from each modalities first go through the kronecker fusion to get an approximation of the joint distribution. We apply self-attention to the features *Z*_*a*_, *Z*_*b*_ to obtain modality-specific ${S}_{a}^{{\prime} }$, ${S}_{b}^{{\prime} }$. Finally we extract the common information by applying cross attention of *Q*_*c*_, ${K}_{a}+{K}_{b}^{{\prime} }$, *V*_*c*_ + *V*_*a*_ + *V*_*b*_ to obtain ${S}_{c}^{{\prime} }$.

We minimize the Mutual Information (MI) loss between concatenated *S*_*a*_ + *S*_*b*_ and ${S}_{c}^{{\prime} }$ to preserve modality-specific information while reducing redundant features. We calculate the variational upper bound called contrastive log-ratio upper bound (vCLUB) as an MI estimator to approximate the intractable MI minimization^74^:

20 $${L}_{v}^{CLUB}(a,b)={{\mathbb{E}}}_{p}(a,b)[\log \,{q}_{\theta }(b| a)]-{{\mathbb{E}}}_{p}(a){{\mathbb{E}}}_{p}(b)[\log \,{q}_{\theta }(b| a)]=\frac{1}{{N}^{2}}{\sum }_{i=1}^{N}{\sum }_{j=1}^{N}[\log \,{q}_{\theta }({b}_{i}| {a}_{i})-\log \,{q}_{\theta }({b}_{j}| {a}_{i})]$$

We use an MLP $q| \theta (b| a)$ to variationally approximate ${p}_{\theta }(b| a)$, which can be optimized maximizing the log-likelihood^73^:

21 $${L}_{{\rm{estimator}}}(a,b)=\frac{1}{N}\mathop{\sum }\limits_{i=1}^{N}\log \,{q}_{\theta }({b}_{i}| {a}_{i})$$

Thus the mutual information loss is defined as:

22 $${\rm{MILOSS}}={L}_{v}^{CLUB}({S}_{a}+{S}_{b})+{L}_{{\rm{estimator}}}({S}_{a}+{S}_{b},{S}_{c})$$

In order not to risk over-suppressing information, we add an additional lambda as hyperparameter to regularize the MI loss. This entails a final loss as the sum of objective loss (MSE for dengue prediction) and regularized MI loss of:

23 $${{\rm{Loss}}}_{{\rm{final}}}={L}_{{\rm{objective}}}(g({h}_{{\rm{final}}}))+\lambda {\rm{MI}}({\rm{concat}}({S}_{a},{S}_{b}),{S}_{c})$$

### Static data scenario for poverty and access to school prediction

To test the value of the satellite image embeddings in simpler tasks, a Support Vector Machine algorithm was trained to predict poverty, and access to school.

A baseline model was created using all the metadata excluding the label, to predict Poverty and Education using 80% of the municipalities to train, and 20% to test as can be seen in the Fig. 2b. Then the embeddings were concatenated to the metadata using the corresponding city, and this resulting dataset was used to train the model in the same tasks to show the improvement when satellite images embeddings were added.

## Data Availability

The dataset used in this paper is available in Physionet under the name A Multi-Modal Satellite Imagery Dataset for Public Health Analysis in Colombia^26^. We also provide a framework called satellite extractor that provides users with a high degree of customization for their specific needs, so that users can download images at a desired timestamp and geographical location: • **Customizing Regions of Interest:** Users can define their own regions of interest within the dataset. This means they can focus on specific geographical areas that are relevant to their research or analysis. This could be a particular city, a region, or even an entire country. This feature allows users to tailor the dataset to their specific needs and ignore irrelevant data. For this procedure, the user should only identify the center coordinates of the region of interest, and then satellite.extractor will define a bounding box with an approximate size of 750 × 750 pixels. • **Improving Satellite Image Quality and Handling Cloud Interference:** To reduce cloud interference and get rid of all black images that are created during data acquisition, users can choose to use a single artifact removal technique or a forward-backward artifact removal approach. Additionally, the code acts as a basis, enabling users to customize the bands that are downloaded based on their own needs. • **Adjusting Image Frequency:** The satellite.extractor package provides users with the ability to modify the frequency of image capture based on a specific timestamp. This feature gives users the power to dictate the regularity of new image acquisition. For example, if a user is tracking temporal changes in vegetation, they might configure the system to procure a new image every month. This process can be similarly applied to other indicators such as urban expansion or changes in weather patterns. The default setting for the satellite.extractor tool is to use the Epiweek system, which operates on a 7-week cycle. However, this can be adjusted to accommodate higher frequencies as per the user’s requirements. • **Setting Time Limits:** Users can also set time limits for the process. This could be useful in scenarios where the user is interested in studying a phenomenon over a specific period. For example, if a user wants to study the impact of a policy change over a year, they can set the time limit to collect data for exactly one year. The satellite images in the SatelliteBench dataset are stored in TIFF format, and each image contains 12 bands. Each image is associated to a file in CSV format with the metadata of the image. The structure of the dataset follows a well-organized directory format, enhancing accessibility and ease of use. Here are the key details regarding the image format and organization: • TIFF Image Format: The satellite images are stored in the Tagged Image File Format (TIFF). Each image comprises 12 bands, providing a multi-spectral representation of the captured data. • Directory Structure: Images are organized within directories, and each directory corresponds to a specific municipality identified by a unique Municipality Code. The Municipality Code serves as a geographical identifier, linking the images to specific locations within Colombia. We have available two subsets: a subset with the top 10 municipalities with most dengue cases, and another full subset with the top 81 municipalities with most dengue cases. • Temporal Identifier - Epi Week: The images are named using the date of capture, allowing for easy identification of the temporal aspect. The date of the image serves as a unique identifier, aligning with the concept of epidemiological weeks (Epi Weeks). • CSV File for Metadata: In the main directory is placed a CSV file that contains essential metadata associated with the image. The CSV file includes information crucial for understanding the context of the images. The CSV file serves as a comprehensive data source, containing details such as Municipality Code, Epi Week, Image Path, Static Data (socioeconomic context), Multi-Class Labels (case status for dengue), and Continuous Data (cases, climatic data).

## Code availability

The source code employed in this study is accessible on Github at the following repository:

https://github.com/mitcriticaldatacolombia/SatelliteBench.

The repository contains all the scripts, methods, and essential files necessary to replicate the experiments and analyses presented in our Nature study. Additionally all the pre-trained models used to prepare this article are available in Physionet and can be downloaded without any restrictions. The full set of images used in this research is also available in Physionet under the name A Multi-Modal Satellite Imagery Dataset for Public Health Analysis in Colombia^26^.

**Repository Structure Overview**

**Readme.md File:** This file provides comprehensive instructions for downloading and setting up the environment required for replication. It is strongly recommended to refer to this document before initiating the replication process.

**Main Directories:**

• **Preprocessing/:** Contains Python codes and functions essential for executing the preprocessing steps across all experiments. These codes are fundamental for preparing the data for subsequent analyses.

• **Models/:** Encompasses all Python code related to the creation of models and backbones utilized in the study. Understanding the contents of this directory is crucial for gaining insights into the model architectures employed.

**Main Files:**

• **Train_selfsupervised.ipynb:** A Jupyter notebook encompassing the entire codebase used for training the models to generate embeddings. This notebook is pivotal for understanding the training procedures applied to achieve the embeddings.

• **Embeddings_generation.ipynb:** A Jupyter notebook designed for utilizing the trained model’s backbone to generate a CSV file containing image embeddings. This file is pivotal for comprehending the process of extracting informative embeddings from the trained model.

• **Time_Series_Fusion_Network_Demo.ipynb:** A Jupyter notebook containing the demonstration code to execute the experiments discussed in this paper for the dengue prediction model. This code leverages the proposed time-series multimodal data fusion model and can be executed seamlessly on Google Colab for a simplified implementation.

• **Access_to_water.ipynb & poverty.ipynb:** These Jupyter notebooks encapsulate the codebase for running experiments related to water access and poverty prediction, respectively. Understanding the content of these notebooks is crucial for reproducing and extending the analyses conducted in the study.

**Demo Dataset:** Within the Demo_Data/ directory, researchers can access essential data for quickly testing the predictions presented in the paper. The following files are available:

• **Embeddings_variational_autoencoder_ResNet50V2_224_1024_12Bands.csv & Embeddings_variational_autoencoder_ResNet50V2_224_1024_3Bands.csv:** These files contain embeddings of satellite images for municipalities with the most dengue cases. Extracted using a Variational Autoencoder (VAE) with a ResNet 50 V2 backbone, these embeddings utilize both RGB and all 12 band images. The CSV files include columns such as “Municipality Code” (municipality ID), “Date” (image collection date), and 1024 columns representing vector embeddings.

• **temperature.csv & precipitation.csv:** Two CSV files providing temperature and precipitation values for the aforementioned 10 municipalities. The columns include “LastDayWeek” (date of the last day of the epidemiological week) and 10 columns indicating the respective municipality for the temperature or precipitation values.

• **Metadata_dengue_tabular.csv:** A CSV file encompassing all metadata and labels used for predictions. The “Municipality code” column specifies the municipality from which the data originates.

Note on Dataset Format:

The dataset employed in this study spans 81 municipalities in Colombia from 2016 to 2018, resulting in a total of 12,636 satellite images. The metadata associated with these images encompasses static sociodemographic variables, indices of poverty, and access to education. Additionally, dynamic epidemiological and climatic metadata were collected, providing a comprehensive spatiotemporal context for the analyses.

Researchers are encouraged to refer to the documentation within the repository for detailed guidance on replicating experiments and understanding the nuances of the dataset.
